# Sulfation of the FLAG epitope is affected by co-expression of G protein-coupled receptors in a mammalian cell model

**DOI:** 10.1038/srep27316

**Published:** 2016-06-07

**Authors:** Morag Rose Hunter, Natasha Lillia Grimsey, Michelle Glass

**Affiliations:** 1 Department of Pharmacology and Clinical Pharmacology, University of Auckland, Auckland, New Zealand

## Abstract

G protein-coupled receptors (GPCRs) are important therapeutic targets and therefore extensively studied. Like most transmembrane proteins, there has been considerable difficulty in developing reliable specific antibodies for them. To overcome this, epitope tags are often used to facilitate antibody recognition in studies on fundamental receptor signalling and trafficking. In our study of cannabinoid CB_1_/dopamine D_2_ interactions we sought to generate HEK293 cells expressing FLAG-tagged D_2_ for use in antibody-based assays of GPCR localisation and trafficking activity, however observed that stable FLAG-hD_2_ expression was particularly challenging to maintain. In contrast, when expressed in cell lines expressing hCB_1_ robust and stable FLAG-hD_2_ expression was observed. We hypothesised that co-expression of CB_1_ might stabilise surface FLAG-hD2 expression, and therefore investigated this further. Here, we describe the observation that co-expression of either cannabinoid CB_1_ or CB_2_ receptors in HEK293 decreases the sulfation of a FLAG epitope appended at the N-terminus of the dopamine D_2_ receptor. Sulfation alters epitope recognition by some anti-FLAG antibodies, leading to the detection of fewer receptors, even though expression is maintained. This demonstrates that cannabinoid receptor expression modifies posttranslational processing of the FLAG-hD_2_ receptor, and importantly, has wider implications for the utilisation and interpretation of receptor studies involving epitope tags.

G protein-coupled receptors (GPCRs) are a large family of proteins which are found embedded into cellular membranes, typically on the cell surface. The general structure of GPCRs is well conserved, with an extracellular N-terminal tail, seven transmembrane alpha-helices joined by intra- and extra-cellular loops, an intracellular eighth helix, and an intracellular C-terminal tail^1^. As their name suggests, GPCRs activate G proteins by acting as a cofactor for the exchange of GDP to GTP on the Gα subunit^2^.

GPCRs are able to function both as monomers, and in groups of two (dimers) or more (oligomers). Dimers and higher order oligomers may be composed of several different GPCRs (heterodimers, or “mosaics”)^3^,^4^. For most Class A GPCRs, it is unknown whether dimerisation is required for normal function. However, there is extensive description of heterodimer formation and function in mammalian cell systems (reviewed in^5^,^6^). Generally, GPCR heterodimers have a more restricted tissue distribution than their component receptors. Thus, therapeutics focusing on heterodimers may offer the opportunity to selectively target a specific subset of receptors within the body and exploit dimer-specific signalling pathways.

One such heterodimer consists of the cannabinoid receptor 1 (CB_1_) and dopamine receptor 2 (D_2_). There is considerable behavioural evidence that the cannabinoid and dopamine systems interact in the rodent and human brain, affecting motor functioning and the reward pathway^7^. CB_1_ and D_2_ are co-localised in GABAergic synapses in the prefrontal cortex^8^ and the nucleus accumbens^9^.

Although both CB_1_ and D_2_ canonically signal through Gαi pathways, this changes to an apparently Gαs signalling pathway when the receptors are co-stimulated in medium spiny neurons, which endogenously express both CB_1_ and D_2_^10^. This signalling switch could be replicated in Human Embryonic Kidney cells (HEK293)^11^, and has been found to be dependent on the co-expression of these two receptors^12^, leading to the hypothesis that this was due to a direct physical interaction between the two receptors - i.e. heterodimerisation. Results consistent with heterodimerisation have been demonstrated by co-immunoprecipitation experiments^11^,^13^, fluorescence resonance energy transfer^14^,^15^,^16^ and bimolecular fluorescence complementation^17^. Furthermore, in medium spiny neurons, knockdown of either CB_1_ or D_2_ receptors reduced the expression of the other^18^, suggesting that protein levels are closely controlled by the activity of both receptors.

In our study of CB_1_/D_2_ interactions we sought to generate HEK293 cell lines expressing FLAG-tagged human (h) D_2_ for use in antibody-based assays of GPCR localisation and trafficking activity, however we observed that stable FLAG-hD_2_ expression was particularly challenging to maintain. When introduced alone, the long-term maintenance of a HEK293 cell line with measurable FLAG-hD_2_ expression proved apparently impossible. While we could transiently express the FLAG-hD_2_ construct easily in HEK293 wildtype cells, expression (as measured by antibody labelling) was very low immediately following antibiotic selection. However, we were interested to note that HEK293 cell lines which also expressed introduced hCB_1_ (with a triple HA tag “3HA”) exhibited robust FLAG-hD_2_ expression and stable lines were established with relative ease. We hypothesised that co-expression of the 3HA-hCB_1_ receptor might stabilise surface FLAG-hD_2_ expression, and therefore investigated this further.

## Results

### Antibody detection of FLAG-hD_2_ throughout the establishment of stable cell lines

In order to investigate whether FLAG-hD_2_ expression was facilitated by co-expression of hCB_1_, HEK293 cell lines (hereafter “HEK”) were transfected with the FLAG-hD_2_ pcDNA3.1+ plasmid and subjected to antibiotic selection to generate stable cell lines. The parental cell lines into which FLAG-hD_2_ was transfected were HEK wildtype (wt), or HEK stably transfected with either 3HA-hCB_1_ or 3HA-hCB_2_. A subset of transfected cells were also cultured without antibiotic selection. Antibody labelling was measured every second passage for 56 days in order to monitor FLAG-hD_2_ expression over time. A clonally-isolated positive control cell line, already characterised in our laboratory as expressing both 3HA-hCB_1_ and FLAG-hD_2_ (i.e., the expected result of the HEK 3HA-hCB_1_+ FLAG-hD2 transfection condition), was used as a labelling control, as this had already been demonstrated to exhibit anti-FLAG antibody labelling.

**Figure 1:**
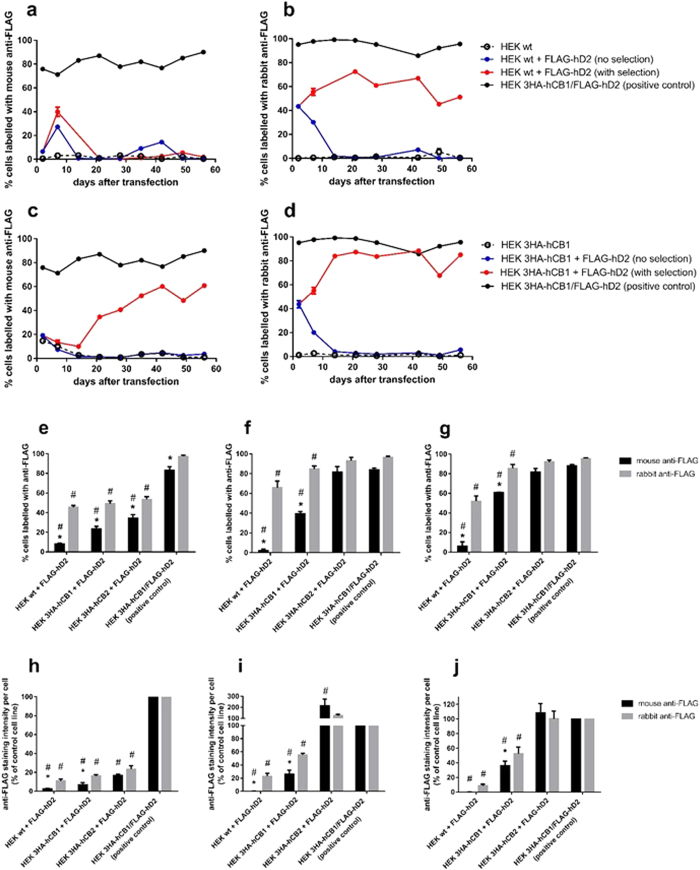
Immunocytochemical analysis of cells exhibiting cell surface FLAG-hD_2_ labelling following transfection into different HEK cell backgrounds, as detected by two anti-FLAG antibodies; time course post-transfection. FLAG-hD_2_ was transfected into HEK wt (**a,b,e,h**), HEK 3HA-hCB_1_ (**c,d,f,i**), or HEK 3HA-hCB_2_ (**g,j**) cell backgrounds. Surface receptors were stained with either mouse anti-FLAG M2 monoclonal (**a,c**), or rabbit anti-FLAG polyclonal (**b,d**) antibodies for 56 days after transfection, with specific analysis at 2 days (transient expression phase; **e,h**), 21 days (immediately after stable selection; **f,i**), and 56 days (after continuous maintenance in selection antibiotic; **g,j**). Images were taken by automated microscopy at 10x magnification and the resultant images were analysed for the proportion of cells positive for anti-FLAG labelling (**a–g**), and the labelling intensity (**g–j**) were analysed. Representative data (one of three independent experiments) showing the time-course of anti-FLAG labelling intensity for the experimental samples (red) compared to that of the untransfected parental cell line (dashed lines), cells transfected but not subjected to antibiotic selection pressure (blue), and a clonal control cell line stably expressing 3HA-hCB_1_ and FLAG-hD_2_ (black) (A–D). Combined data (mean + SEM) from three independent experiments comparing anti-FLAG labelling patterns at key time points (**e–j**). Intensity analysis is normalised to matched labelling of untransfected HEK wt cells (0%) and the HEK 3HA-hCB1/FLAG-hD2 control cell line (100%) (**h–j**). p < 0.05, *significant difference within cell line labelled with rabbit anti-FLAG versus mouse anti-FLAG; ^#^significant difference compared to HEK 3HA-hCB1/FLAG-hD_2_ control cell line with same antibody.

Utilising a mouse monoclonal anti-FLAG antibody, striking differences in the apparent proportion of cells expressing surface FLAG-hD_2_ were observed depending on the cell background utilised. Following transfection into HEK wt, transient FLAG-hD_2_ expression was detected during the first 10 days of culture, however the proportion of FLAG-hD_2_ positive cells subsequently decreased during antibiotic selection (which took approximately 14–18 days, as determined by the death of all cells in a flask of untransfected cells), after which FLAG labelling was nearly completely lost (Fig. 1a,f,g). Conversely, when transfected into a previously established HEK 3HA-hCB_1_ line^19^ detection of FLAG-hD_2_ was retained during the selection phase, and the proportion of expressing cells tended to increase with continued selection pressure, as is typically expected on the establishment of a cell line stably expressing the introduced gene of interest (Fig. 1c,f,g). A similar pattern was observed when FLAG-hD_2_ was transfected into a 3HA-hCB_2_-expressing HEK cell line, where the ability to detect FLAG-hD_2_ was retained with long term culture (Fig. 1e–g). We also noted that only approximately 80% of the positive-control HEK FLAG-hD_2_/3HA-hCB_1_ cell line was indicated to express FLAG-hD_2_, even though this line was derived by clonal isolation.

To validate these findings, and to permit co-labelling with mouse anti-HA antibody to detect 3HA-hCB_1_, we performed parallel immunocytochemistry experiments on the same cells as assayed above with a polyclonal rabbit anti-FLAG antibody. Surprisingly, labelling with this antibody indicated approximately equivalent proportions of FLAG-positive cells between the HEK wt, 3HA-hCB_1_ and 3HA-hCB_2_ cell backgrounds (Fig. 1b,d,e–g). After long-term culture (day 56, 16 passages post-transfection) the proportion of cells labelled with rabbit anti-FLAG remained approximately unchanged when compared to day 21, regardless of the parental cell line (comparison of rabbit anti-FLAG labelling on day 21 versus day 56 in HEK wt, HEK 3HA-hCB_1_, and HEK 3HA-hCB_2_; p > 0.05), although overall more cells were labelled with rabbit anti-FLAG antibody when the cell background contained 3HA-hCB_1_ or 3HA-hCB_2_, as compared with HEK wt cells (85 ± 3%, 93 ± 4% and 66 ± 7%, respectively; Fig. 1b,d–g). This antibody indicated that close to 100% of the positive-control HEK FLAG-hD_2_/3HA-hCB_1_ cells expressed FLAG-hD_2_, as was expected due to the clonal nature of this line.

Importantly, under the conditions utilised in these experiments neither anti-FLAG antibody labelled the untransfected parental cell lines, indicating a lack of non-specific binding (Fig. 1a–d).

The intensity of antibody labelling was also compared across the transfection conditions. Using the same microscopy images as were analysed to measure the proportion of positively stained cells, the total intensity of antibody labelling above background was averaged across all cells in the image. Since each anti-FLAG antibody provides a different absolute level of staining, labelling was normalised so that untransfected HEK wt cells were set to 0%, and the HEK 3HA-hCB_1_/FLAG-hD_2_ control line was set to 100% labelling for each anti-FLAG antibody.

The intensity analysis demonstrated differences in the degree of anti-FLAG labelling between the parental cell lines, but overall was in agreement with the prior measured proportions of positive cells. On day 2, in the transient transfection phase, there was low average FLAG-hD_2_ labelling (Fig. 1h). This is likely a reflection of the small proportion of cells which were initially transfected. By day 21, at the conclusion of antibiotic selection, the transfected HEK wt cell was essentially devoid of mouse anti-FLAG labelling, whereas a moderate degree of FLAG-hD_2_ labelling was detected in the 3HA-hCB_1_ line with this antibody (Fig. 1i). By comparison, the rabbit anti-FLAG antibody produced significantly more labelling than the mouse antibody. Though the indicated labelling per cell was considerably lesser than that of the positive control line, this was likely a result of these lines being mixed populations comprised of cells with a range of expression levels as opposed to the clonal HEK 3HA-hCB_1_/FLAG-hD_2_ control. Interestingly, FLAG-hD_2_ labelling by both antibodies was considerably higher in the HEK 3HA-hCB_2_ cell background than in the HEK 3HA-hCB_1_ background.

Due to the markedly different labelling observed with the rabbit anti-FLAG and mouse anti-FLAG antibodies, binding and functional studies were carried out as alternative means to determine FLAG-hD_2_ expression in the cell lines generated.

### Determination of D_2_ expression by radioligand binding

**Figure 2:**
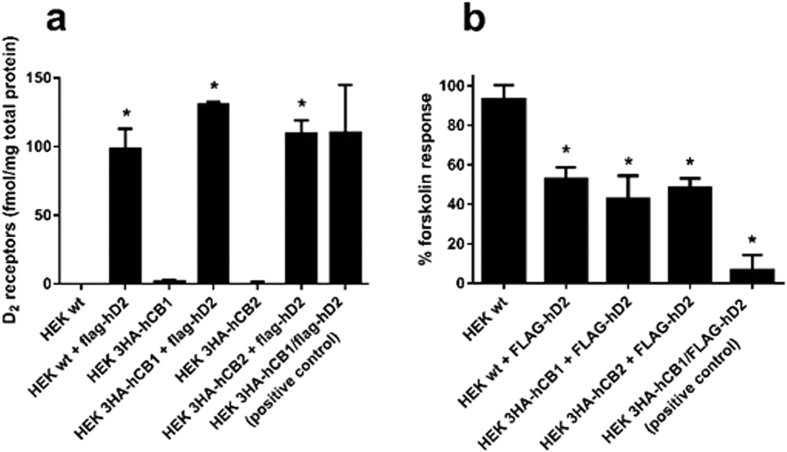
[^3^H]-raclopride whole-cell binding and cAMP signalling in HEK cell lines stably transfected with FLAG-D_2_. Cells were stably transfected with FLAG-hD_2_ and assayed day 56-57/16 passages post-transfection. [^3^H]-raclopride binding assays were performed, demonstrating approximately equal numbers of D_2_ binding sites between all FLAG-hD_2_-transfected cell lines, but not in untransfected cell lines (**a**). Mean ± SEM of three independent experiments, *p < 0.05 significant compared to untransfected parental cell line. The ability of 100 nM quinpirole to inhibit 5 μM forskolin-induced cAMP increase was measured using a cAMP biosensor over 15 minutes of stimulation in cell lines 57 days/16 passages post-transfection with FLAG-hD_2_ (other than “HEK wt” which was not transfected) (**b**). cAMP responses were normalised to forskolin alone (100%) and vehicle (0%). Mean + SEM of three independent experiments. p < 0.05; *significant inhibition compared to forskolin alone.

To determine receptor number, whole cell radioligand binding assays were performed 56–57 days (16 passages) post-transfection with [^3^H]-raclopride, a D_2_-selective antagonist. All cells transfected and selected for FLAG-hD_2_ expression contained approximately equivalent receptor levels of 98–131 fmol D_2_ receptors per mg of total cell protein (no statistically significant differences; Fig. 2a). Negligible specific binding was detected the cell lines not transfected with hD_2_, indicating that it was the introduced FLAG-hD_2_ transgene which conferred [^3^H]-raclopride binding in these samples.

### Demonstration of FLAG-hD_2_ functionality by cAMP signalling assay

To determine receptor functionality, cAMP assays were performed 57 days/16 passages post-transfection with quinpirole, a D_2_-selective agonist. As shown in Fig. 2b, all transfected cells were responsive to quinpirole (100 nM) as indicated by a reduction in cAMP relative to forskolin alone (100%) and as expected for a Gαi-coupled receptor. FLAG-hD_2_-transfected cell lines which had originally contained 3HA-hCB_1_ or 3HA-hCB_2_ retained their responsiveness to CP55,940, a non-selective CB_1_/CB_2_ agonist (data not shown). All three experimental cell lines trended towards less responsiveness to quinpirole as compared to the HEK 3HA-hCB_1_/FLAG-hD_2_ positive control cell line. Though this difference was not statistically significant, this apparent lesser responsiveness was not unexpected given that the experimental cell lines were mixed populations with a range of receptor levels, compared to the clonal, high expressing HEK 3HA-hCB_1_/FLAG-hD_2_ cell line. However, the newly-generated FLAG-hD_2_-transfected cell lines exhibited an equivalent degree of D_2_-mediated signalling, regardless of whether hCB_1_, hCB_2_ or neither were expressed when hD_2_ was introduced.

### Determination that antibody recognition is sensitive to sulfation

Having observed a stark contrast between the results of antibody labelling experiments (which suggested highly variable FLAG-hD_2_ expression between transfection conditions) and the radioligand binding and cAMP results (which indicated similar D_2_ expression in all transfection conditions), we hypothesised that the FLAG antibodies were differentially sensitive to a feature of the FLAG epitope which was present when D_2_ was expressed alone, but not when expressed with CB_1_ or CB_2_. The tyrosine residue in FLAG has previously been reported as being sulfated^20^,^21^, therefore we treated cells with sodium chlorate to inhibit sulfation^22^. Treated and untreated cells were then stained for cell surface FLAG epitope, and imaged by automated microscopy as above.

**Figure 3:**
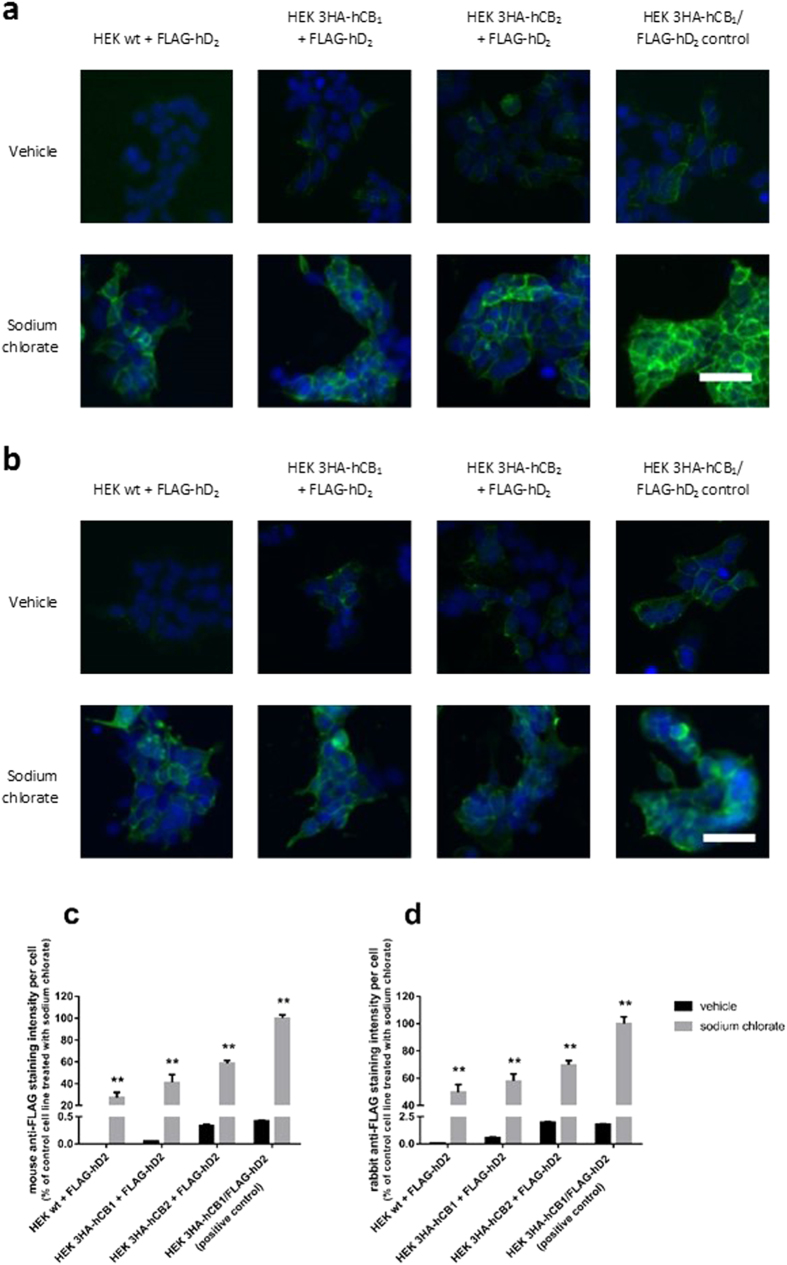
Antibody labelling with two anti-FLAG antibodies with and without inhibition of sulfation with sodium chlorate. Cells were treated with vehicle or the sulfation inhibitor sodium chlorate, and labelled with either mouse anti-FLAG (**a,c**) or rabbit anti-FLAG (**b,d**) antibodies. Images were taken by automated microscopy at 10× magnification. Representative images of three independent experiments; all images taken under equivalent conditions (**a,b**). Antibody labelling (green), and Hoechst 33258 to stain nuclei (blue); scale bar, 50 μm. The intensity of antibody labelling was quantified, and displayed relative to the HEK 3HA-hCB_1_/FLAG-hD_2_ control cell line (**c,d**). Representative data (mean + SEM) from one of three independent experiments performed in triplicate; all differences between vehicle and sodium chlorate treated cells are statistically significant (**p < 0.01).

Vastly greater anti-FLAG labelling intensity was found in cells treated with sodium chlorate (Fig. 3). We verified that this treatment did not affect the nonspecific labelling of either antibody on untransfected cells. Qualitatively, these images demonstrate that anti-FLAG labelling (with both the mouse and rabbit antibodies) was increased in all FLAG-expressing cell lines when sulfation was inhibited using sodium chlorate. Importantly, following sodium chlorate treatment, both FLAG antibodies now indicate essentially equivalent FLAG-hD_2_ expression regardless of the cell background into which they were transfected. As well as now reflecting a consistent antibody labelling pattern between the two antibodies, this finding aligned with our radioligand binding and cAMP functional data that likewise indicated similar expression levels.

## Discussion

Epitope tags are commonly used for experiments requiring immunocytochemical detection of exogenous GPCRs, primarily because antibodies are difficult to raise to endogenous GPCR epitopes^23^,^24^,^25^,^26^ and also to reduce reagent costs. The commercially-available antibodies to epitope tags such as haemagglutinin (“HA”) and DYKXXD (“FLAG”) are well established in the literature, making the use of epitope tags an attractive approach to experiments which would otherwise require the generation and validation of GPCR-specific antibodies.

The hD_2_ construct utilised in this study is N-terminally FLAG-tagged, and indeed “FLAG” has been specifically recommended for receptor internalisation assays^27^,^28^. Anti-FLAG antibodies were employed to label this FLAG-hD_2_ transgene with the aim of measuring relative protein expression. We were interested to observe that co-expression of either 3HA-hCB_1_ or 3HA-hCB_2_ influenced the degree of FLAG-hD_2_ labelling; seemingly indicating that co-expression with cannabinoid receptors may increase hD_2_ surface expression. This finding was re-capitulated with two different FLAG antibodies, though to differing extents.

The two commercially-available anti-FLAG antibodies we compared were polyclonal rabbit anti-FLAG (Sigma, cat. F7425) and monoclonal mouse anti-FLAG M2 (Sigma, cat. F1804). While there was negligible non-specific binding to untransfected cells, an important difference was seen between the ability of the antibodies to detect the FLAG epitope. The mouse anti-FLAG antibody essentially indicated a complete lack of FLAG-hD_2_ expression, *unless* the transgene was introduced into cells already expressing either 3HA-hCB_1_ or 3HA-hCB_2_. Although the rabbit anti-FLAG antibody could detect FLAG-hD_2_ transfected into the HEK wt line, labelling intensity was again greater in the 3HA-hCB_1_ and 3HA-hCB_2_ lines.

This interesting finding of an apparent cannabinoid receptor-induced alteration of hD_2_ expression was, however, called into question by our follow-up experiments examining D_2_-selective radioligand binding and cAMP signalling. These instead indicated that hD_2_ expression was equivalent between the cell backgrounds tested, irrespective of the presence of 3HA-hCB_1_ or 3HA-hCB_2_. Given these measures are a more direct indication of D_2_ expression and functionality than anti-FLAG labelling (and that a prior report had also suggested that D_2_ ligand binding is unaffected by CB_1_ co-expression^11^), we hypothesised that the the fidelity of the anti-FLAG labelling was questionable.

Some antibodies are known to be sensitive to epitope post-translational modifications, including the M2 mouse monoclonal antibody used in this study, which has been reported to be sensitive to sulfation^20^,^21^. Tyrosine sulfation occurs in the Golgi^29^, and is a common posttranslational modification of secreted and transmembrane proteins (reviewed in^30^). Tyrosine sulfation is more likely when the tyrosine residue is surrounded by acidic residues (summarised in^31^), making the FLAG epitope a likely target, as it contains one aspartic acid in the -1 position, and four aspartic acid residues in positions +2 to +5, relative to the tyrosine. Furthermore, the lysine in the +1 position is also consistent with a tyrosine sulfation site, as nearby polar residues are more permissive of this modification^31^. Sulfation of the FLAG epitope has been shown to prevent the mouse anti-FLAG M2 antibody from binding and reduces the binding of other anti-FLAG antibodies^21^,^22^. Indeed, sulfation of the N-terminus has been observed for several GPCRs, including the sphingosine 1-phosphate S1P1 receptor^32^, chemokine CCR2 receptor^22^, and complement component 5a C5aR1 receptor^33^. These previously-reported observations led us to test whether sulfation was a factor in antibody binding in our experimental system.

Cells were treated with sodium chlorate to reduce sulfation activity^22^,^34^,^35^,^36^, resulting in a considerable increase in labelling of both the mouse M2 monoclonal and rabbit polyclonal anti-FLAG antibodies. This is consistent with unmasking of the FLAG epitope by removal of sulfation. These results indicate that the predominant idiotypes in the polyclonal rabbit anti-FLAG are also unable to detect sulfated FLAG-hD_2_, thereby giving inconsistent and potentially misleading results when comparing cell lines or drug treatments where sulfation is occurring differently.

When we look specifically at D_2_, the addition of the FLAG epitope on the D_2_ N-terminus is a relatively common way of monitoring D_2_ receptor expression and trafficking (for example^11^,^37^,^38^,^39^,^40^), usually without a second antibody to compare labelling profiles. Additionally, the mouse M2 anti-FLAG antibody specifically has been used for detection of N-terminally-tagged FLAG-hD_2_^38^,^39^,^40^,^41^, which this study has shown has a high sensitivity to the sulfation state of the FLAG-hD_2_ construct. In light of the results of this study, future work should be designed to avoid or account for this phenomenon.

The results of our stable transfection experiments showed that expression of 3HA-hCB_1_ or 3HA-hCB_2_ modifies the detectability of the FLAG-hD_2_ transgene. CB_1_ and D_2_ receptors are known to form a heterodimer, with many of these studies carried out in HEK293 cells^11^,^13^,^14^,^15^,^17^. It is possible that a physical interaction of CB_1_ and D_2_ while trafficking via the synthetic pathway protects the FLAG epitope in the FLAG-hD_2_ construct from sulfation, perhaps either by altering the conformation of the D_2_ N-terminal tail or by otherwise altering sulfotransferases’ access to the FLAG epitope. When designing these experiments, CB_2_ was chosen as a control cell line because we know of no published evidence suggesting that a CB_2_-D_2_ heterodimer exists. Therefore, it was a surprise to find that 3HA-hCB_2_ expression also altered FLAG-hD_2_ posttranslational modification. Although there are no reported studies describing CB_2_-D_2_ heterodimeric or signalling interactions, GPCRs can form non-physiologically relevant heterodimers in transfected cell lines (for example the dopamine D_1_-D_2_ heterodimer, which appears to have a distinct signalling phenotype but cannot be demonstrated *in vivo*^42^), and this may be occurring in our experimental system. We also observed that FLAG-hD2 was more readily detected by both anti-FLAG antibodies in the HEK 3HA-hCB_2_ parental cell line than in either of the other experimental cell lines, although all cell lines contained equivalent FLAG-hD2 expression. This difference in detection was maintained even after inhibition of sulfation, suggesting that the FLAG epitope was more accessible in this cell line in general. This may have occurred through a different pattern of receptor dimerization, altering the accessibility of the FLAG-hD_2_ N-terminus for antibodies.

Potentially, the similarity in the effects 3HA-hCB_1_ and 3HA-hCB_2_ have on FLAG-hD_2_ expression are due to altered cell signalling, rather than a direct heterodimerisation interaction. Both CB_1_ and CB_2_ receptors signal through Gαi-pathways, have similar functional effects in cAMP and pERK signalling^43^,^44^,^45^, and are known to exhibit constitutive signalling^46^,^47^,^48^,^49^, including in unstimulated HEK cells^19^,^50^. This constitutive signalling may alter the posttranslational modification repertoire of the cell, thus increasing the frequency of sulfation reactions. While we are not aware of any previous reports of signalling altering sulfation, certainly signalling-mediated changes in post-translational modifications have been reported (for example^51^,^52^).

We have demonstrated that in the HEK cell line, antibody detectability of the FLAG epitope in the FLAG-hD_2_ construct is altered when it is co-expressed with HA-tagged cannabinoid receptors, seemingly via inhibiting posttranslational modification of the FLAG epitope. It is unclear whether this is due to specific physical interactions between D_2_ and CB_1_/CB_2_, or an indirect influence of cannabinoid receptors on cellular function which may also affect other GPCRs and proteins. However, sulfation is a fundamental eukaryotic posttranslational modification which could well be influenced by various manipulations or drug treatments. There is therefore considerable potential for FLAG epitope detectability to be influenced unintentionally and this study suggests caution is required when utilising epitope tags, particularly FLAG, for detection of protein expression. Indeed, careful consideration of potential posttranslational modification sites in the target epitope is an important consideration when designing or utilising antibodies in general^53^. In this case our initial hypothesis - that coexpression of CB_1_ could stabilise D_2_ expression, potentially due to dimerization of the receptors - has transpired to be an artefact of the anti-FLAG antibody labelling that is differentially regulated by cannabinoid receptor expression. Given the widespread use of epitope tags in fundamental receptor studies, this finding has far-reaching consequences for the interpretation of these experiments.

## Materials and Methods

### Plasmid construction

The FLAG-hD_2_ pcDNA3.1+ construct was generated by chimerizing the FLAG epitope to the N-terminus of hD_2_ cDNA in pcDNA3.1+ (with the three amino acid linker sequence “EFT” between FLAG and hD_2_, and retaining the start codon of hD_2_; cDNA Resource Centre (www.cdna.org), #DRD0200001). 3HA-hCB_1_ pEF4 A and 3HA-hCB_2_ pEF4A constructs have been described previously^19^,^54^.

### Cell culture

HEK cells were cultured in DMEM with 10% FBS, at 37 °C in 5% CO_2_. Stable cell lines expressing 3HA-hCB_1_ pEF4A and 3HA-hCB_2_ pEF4A constructs were generated by transfection with Lipofectamine 2000 following the manufacturer’s instructions and selected for using 350 μg/ml zeocin. These cells were clonally isolated (HEK 3HA-hCB1) or FACS sorted (HEK 3HA-hCB2) before use in further experiments.

For stable transfection time course experiments, replicate experiments were performed at least one week apart to ensure that time point observations remained independent. HEK wildtype (wt; ATCC #CRL-1573), HEK 3HA-hCB_1_^19^ or HEK 3HA-hCB_2_^54^ cell lines were transfected with FLAG-hD_2_ pcDNA3.1+ plasmid, using Lipofectamine 2000 following the manufacturer’s instructions. Two days after transfection, 550 μg/ml G418 was added to the growth media to select for cells harbouring stably-integrated plasmid. Cell lines were passaged twice-weekly, being allowed to get no more than 90% confluent before passaging, and remaining in 550 μg/ml G418 for the duration of the experiment.

### Immunocytochemistry

Polyclonal anti-FLAG antibody (raised in rabbit) was obtained from Sigma-Aldrich (MO, USA; cat. F7425; lot numbers 001M4789 and 093M4798) and used at a 1:400 or 1:500 dilution respectively, depending on batch. Monoclonal “M2” anti-FLAG antibody (raised in mouse) was also obtained from Sigma-Aldrich (cat. F1804; lot numbers SLBH1191V and 080M6035), and used at approximately 2 ug/ml. Secondary antibodies were obtained from Molecular Probes, Life Technologies (CA, USA): anti-mouse Alexa 488 (cat. A11029), anti-mouse Alexa 594 (cat. A11032), anti-rabbit Alexa 594 (cat. A11037), all raised in goat and used at a 1:400 dilution.

Cells were seeded in poly-L-lysine or poly-D-lysine treated clear 96-well plates, at an appropriate density to ensure 50–80% confluency at the time of antibody application. When required, cells were grown in 50 mM sodium chlorate (in standard growth media) for 48 hours before immunocytochemistry. Primary antibody labelling was performed on live cells, allowing selective labelling of the surface receptor population. Cells were equilibrated with assay media (DMEM + 5 mg/ml BSA) for 30 minutes, then incubated with primary antibodies diluted in assay media for 30 minutes at 37 °C to label surface receptors. Cells were then washed twice with assay media before fixation with 50% methanol + 50% acetone at 4 °C for 10 minutes. Secondary antibodies were applied after fixation, and diluted in phosphate buffered saline containing 1% normal goat serum, 0.2% Triton X-100, and 0.4 mg/ml thiomersal. Finally, DNA was stained with Hoechst 33258 in phosphate buffered saline containing 0.2% Triton X-100.

Images were acquired using the ImageXpress Micro XLS (Molecular Devices, CA, USA) automated microscope, at 10× magnification, with four sites imaged per well. To analyse the proportion of cells exhibiting antibody labelling above background noise, images were analysed using the MetaXpress (version 5.3.0.4, Molecular Devices) “Multiwavelength cell scoring” function, or the MetaMorph (versions 6 and 10; Molecular Devices) “Cell scoring” function. These functions identify each cell by nuclear Hoechst staining, followed by segmentation of one or both wavelengths of interest in a user-defined “nucleus and cytoplasm” area.

To analyse the average intensity of labelling across the total population of cells, images were processed using MetaMorph software (versions 6 and 10), using the “Total Grey Value Per Cell” method described by Grimsey, *et al*.^55^ with some modifications^56^. This analysis paradigm measures the total intensity of fluorescent antibody labelling above background and averages this intensity of antibody labelling between the total number of nuclei counted per image, thereby provideing labelling intensity results as a population average.

### Radioligand binding

Cells were seeded in poly-D-lysine treated 24-well plates and grown until 50–80% confluent. Cells were then incubated for 30 minutes at 37 °C with assay buffer (DMEM + 5 mg/ml BSA), followed by 30 minutes at 37 °C with [^3^H]-raclopride (Perkin Elmer, MA, USA) at 1.3 nM, with or without 10 μM unlabelled raclopride as a displacer. Assay buffer was removed and cells were washed twice in ice-cold phosphate buffered saline, then lysed in 0.1 M NaOH for 10–20 minutes. Lysate samples were then mixed with scintillation fluid and scintillation events were measured for 3 minutes per sample in a Wallac 1450 MicroBeta TriLux (Perkin Elmer, MA, USA). Lysate samples were also assayed in DC protein assay (Biorad, CA, USA) to enable normalisation to protein concentration.

### cAMP signalling assay

Cells were transiently transfected using Lipofectamine 2000 with V8-CAMYEL, a bioluminescence resonance energy transfer (BRET)-based cAMP biosensor^57^. 48 hours after transfection, cells were equilibrated with assay buffer (HBSS + 1 mg/ml BSA) for 30 minutes, and then incubated for a further 5 minutes with the luciferase substrate coelenterazine h to a final concentration of 5 μM. Forskolin (an adenylate cyclase agonist) and quinpirole (a selective D_2_ agonist) were then added, and luminescence was detected immediately in a VictorXLight plate reader (Perkin Elmer, MA, USA), using 460/25 nm and 535/25 nm filter sets, with temperature control at 37 °C.

An inverse BRET ratio was calculated by dividing the donor signal by the acceptor signal, such that higher BRET ratio reflects greater cytoplasmic cAMP concentration. Inverse BRET ratio responses were measured for 15 minutes, and plotted as a function of time. The overall response over time was determined by an area under the curve calculation, representing the cumulative cAMP response. Data was processed and graphed using Prism (version 6, GraphPad, CA, USA), and agonist responses normalised to Vehicle (0%) and forskolin (100%).

### Data analysis

Data were plotted and statistical analysis was performed using GraphPad Prism (version 6, GraphPad, CA, USA). The Brown-Forsythe test for equal variance was performed to ensure parametric testing was appropriate. Paired/repeated measures testing was performed in all experimental designs where matching was applicable. Results were analysed with one-way analysis of variance (ANOVA) with Tukey’s multiple comparisons post-hoc testing, or two-way ANOVA with Sidak’s multiple comparisons, as appropriate.

## Additional Information

**How to cite this article**: Hunter, M. R. *et al*. Sulfation of the FLAG epitope is affected by co-expression of G protein-coupled receptors in a mammalian cell model. *Sci. Rep.* **6**, 27316; doi: 10.1038/srep27316 (2016).
